# Homeobox B9 integrates bone morphogenic protein 4 with inflammation at atheroprone sites

**DOI:** 10.1093/cvr/cvz235

**Published:** 2019-08-29

**Authors:** Celine Souilhol, Ismael Gauci, Shuang Feng, Blanca Tardajos Ayllon, Marwa Mahmoud, Lindsay Canham, Maria Fragiadaki, Jovana Serbanovic-Canic, Victoria Ridger, Paul Charles Evans

**Affiliations:** Department of Infection, Immunity and Cardiovascular Disease, Bateson Centre for Lifecourse Biology, INSIGNEO Institute for Cardiovascular Medicine, Faculty of Medicine Dentistry and Health, Beech Hill Road, University of Sheffield, Sheffield S10 2RX, UK

**Keywords:** Endothelial, Shear stress, Hox, BMP4, NF-κB

## Abstract

**Aims:**

Atherosclerosis develops near branches and bends of arteries that are exposed to disturbed blood flow which exerts low wall shear stress (WSS). These mechanical conditions alter endothelial cells (EC) by priming them for inflammation and by inducing turnover. Homeobox (Hox) genes are developmental genes involved in the patterning of embryos along their anterior–posterior and proximal–distal axes. Here we identified Hox genes that are regulated by WSS and investigated their functions in adult arteries.

**Methods and results:**

EC were isolated from inner (low WSS) and outer (high WSS) regions of the porcine aorta and the expression of Hox genes was analysed by quantitative real-time PCR. Several Hox genes (HoxA10, HoxB4, HoxB7, HoxB9, HoxD8, HoxD9) were significantly enriched at the low WSS compared to the high WSS region. Similarly, studies of cultured human umbilical vein EC (HUVEC) or porcine aortic EC revealed that the expression of multiple Hox genes (HoxA10, HoxB9, HoxD8, HoxD9) was enhanced under low (4 dyn/cm^2^) compared to high (13 dyn/cm^2^) WSS conditions. Gene silencing studies identified Hox genes (HoxB9, HoxD8, HoxD9) that are positive regulators of inflammatory molecule expression in EC exposed to low WSS, and others (HoxB9, HoxB7, HoxB4) that regulated EC turnover. We subsequently focused on HoxB9 because it was strongly up-regulated by low WSS and, uniquely, was a driver of both inflammation and proliferation. At a mechanistic level, we demonstrate using cultured EC and murine models that bone morphogenic protein 4 (BMP4) is an upstream regulator of HoxB9 which elicits inflammation via induction of numerous inflammatory mediators including TNF and downstream NF-κB activation. Moreover, the BMP4-HoxB9-TNF pathway was potentiated by hypercholesterolaemic conditions.

**Conclusions:**

Low WSS induces multiple Hox genes that control the activation state and turnover of EC. Notably, low WSS activates a BMP4-HoxB9-TNF signalling pathway to initiate focal arterial inflammation, thereby demonstrating integration of the BMP and Hox systems in vascular pathophysiology.

## Graphical Abstract

**Graphical Abstract:**
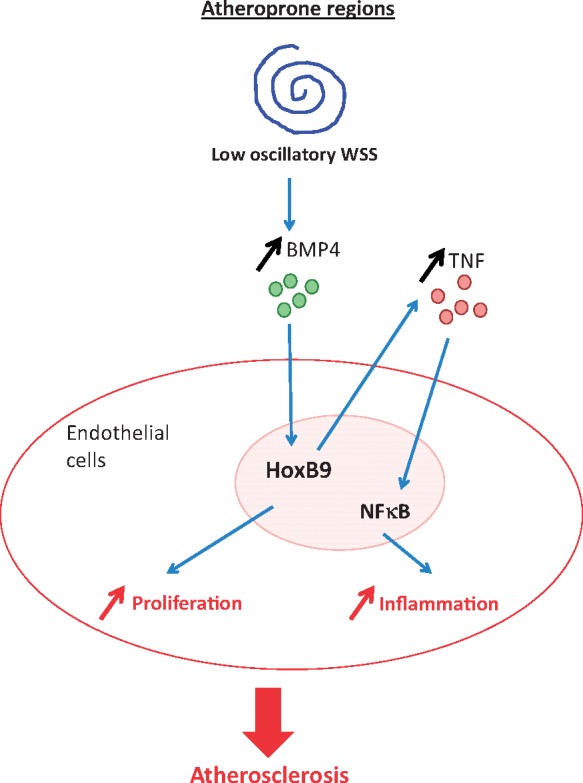

## 1. Introduction

Although associated with risk factors that act systemically (e.g. hypercholesterolaemia, smoking, age), atherosclerosis develops preferentially near branches and bends of arteries.

^1^ ^,^ ^2^ Disturbed blood flow at these sites generates low wall shear stress (WSS)^3^^,^^4^ which promotes atherogenesis by inducing vascular inflammation^4–6^ and endothelial cell (EC) turnover^7^^,^^8^ coupled to enhanced permeability to cholesterol-containing lipoproteins.^9^ Low WSS also correlated with plaque burden in patients with coronary artery disease,^10^^,^^11^ and in animal models where low WSS promoted the development of inflamed lesions with features of vulnerability.^12^^,^^13^ By contrast, regions of arteries that are exposed to uniform flow generating high time-averaged WSS are protected from inflammation and atherosclerosis. Focal atherosclerosis is driven by bone morphogenic protein 4 (BMP4) which is up-regulated by low oscillatory WSS and down-regulated by high WSS in cultured EC,^14^ and correspondingly is enriched at sites of low WSS in the murine aorta and in EC overlying human coronary artery plaques.^14^^,^^15^ At a functional level, BMP4 induces EC expression of VCAM-1 and ICAM-1^14–16^ which elicit vascular inflammation by capturing circulating leucocytes from the bloodstream to the vessel wall. Here we investigated the mechanisms that couple BMP4 to EC activation and inflammation and found that it signals via a member of the Homeobox (Hox) family.

Hox transcription factors were discovered in *Drosophila melanogaster*^17^ and shown to regulate multiple developmental processes in both invertebrates and vertebrates.^18^ The human genome contains 39 HOX genes that localize to four clusters in the genome (called HOX A, B, C, and D). They are expressed at distinct regions of the embryo and act as master regulators of segmental identity and body patterning by activating transcriptional programmes that control cell fate decisions.^19^ Notably, the expression of several Hox gene varies according to the anatomy of the vascular tree.^20–26^ For example, HoxA1 expression is enhanced in the aortic arch which is relatively prone to atherosclerosis compared to the thoracic aorta which is atheroresistant,^22^ whereas HoxA4 exhibits the opposite pattern.^23^ Although some Hox genes exhibit a distinct pattern of expression in arteries, their potential function in arterial EC and contribution to atherosusceptibility has not been studied.

Here we identified several Hox genes that were enriched at a low WSS region of the aorta. Gene silencing studies revealed that Hox genes are necessary for inflammation, EC proliferation, and EC apoptosis in EC exposed to low WSS. Detailed mechanistic studies revealed a BMP4-HoxB9 pathway that primed EC exposed to low WSS for inflammation by inducing the expression of multiple inflammatory mediators, receptors, and signalling molecules.

## 2. Methods

### 2.1 Materials

BMP4 and anti-TNF blocking antibodies were obtained commercially (Supplementary material online, *Table S1*). DMH1 and LDN193189 were obtained from Sigma.

### 2.2 Isolation of EC, culture, and exposure to WSS

Pig aortas from 4 to 6 months old animals (weight approximately 80 kg) were obtained immediately after slaughter from a local abattoir. They were cut longitudinally along the outer curvature to expose the lumen. Porcine aortic EC (PAEC) exposed to high (outer curvature) or low (inner curvature) WSS were harvested using collagenase (1 mg/mL for 10 min at room temperature) prior to gentle scraping for analysis by quantitative real-time PCR (qRT-PCR). For cell culture experiments, PAEC were isolated using collagenase digestion and cultured on 1% gelatin using M199 growth medium supplemented with foetal bovine serum (20%), L-glutamine (4 mmol/L), EC growth supplement (30 µg/mL), penicillin (100 U/mL), streptomycin (100 µg/mL), and heparin (10 IU/mL). Human umbilical vein EC (HUVEC) were purchased from PromoCell and cultured according to the manufacturer’s recommendations. EC at passage 3–5 were cultured until confluent in 6-well plates and exposed to flow using an orbital shaking platform (PSU-10i; Grant Instruments) housed inside a cell culture incubator. The radius of orbit of the orbital shaker was 10 mm and the rotation rate was set to 210 rpm. This motion caused swirling of the culture medium over the cell surface generating low WSS (approximately 5 dynes/cm^2^) with varied directionality at the centre and high (approximately 11 dynes/cm^2^) WSS with uniform direction at the periphery. Alternatively, HUVEC were cultured on Ibidi gelatin-coated µ-Slides (Ibidi GmbH) until they reached confluency. Flowing medium was then applied using the Ibidi pump system to generate low (4 dyn/cm^2^), low oscillatory (±4 dyn/cm^2^, 0.5 Hz), or high (13 dyn/cm^2^) WSS. The slides and pump apparatus were enclosed in a cell culture incubator warmed to 37°C.

### 2.3 Mouse lines

HoxB9^−^^/^^−^ mice (C57BL/6) obtained from Prof. Mario Capecchi,^27^ ApoE^−/−^ (apolipoprotein-E null) mice and wildtype C57BL/6 controls were housed under specific-pathogen free conditions. Some ApoE^−/−^ mice were exposed to a high fat diet containing 21% (wt/wt) fat, 0.15% (wt/wt) cholesterol, and 0.296% (wt/wt) sodium for 6 weeks. Animal care and experimental procedures were carried out under licenses issued by the UK Home Office and local ethical committee approval was obtained. All animal procedures conformed to the guidelines from Directive 2010/63/EU of the European Parliament on the protection of animals used for scientific purposes. Mice between 2 and 3 months of age were used for experimentation. Experiments were carried out using female mice because vascular physiology varies between sexes in mice.

### 2.4 *En face* staining of murine endothelium

The expression levels of specific proteins were assessed in EC at regions of the inner curvature (susceptible site) and outer curvature (protected site) of murine aortae by *en face* staining. Animals were killed by intraperitoneal injection of pentobarbital (40 mg) and aortae were perfused *in situ* with PBS (at a pressure of approximately 100 mm Hg) and then perfusion-fixed with 4% paraformaldehyde prior to harvesting. Fixed aortae were tested by immunostaining using specific primary antibodies (Supplementary material online, *Table S1*) and Alexafluor568-conjugated or Alexafluor488-conjugated secondary antibodies. EC were identified by co-staining using anti-CD31 or anti-CDH5 antibodies. Nuclei were identified using a DNA-binding probe with far-red emission (To-Pro-3). Stained vessels were mounted prior to visualization of endothelial surfaces *en face* using confocal laser-scanning microscopy (Zeiss LSM510 NLO inverted microscope). Isotype-matched monoclonal antibodies raised against irrelevant antigens or pre-immune rabbit sera were used as experimental controls for specific staining. The expression of particular proteins at each site was assessed by quantification of fluorescence intensity for multiple cells (at least 50 per site) using Image J (1.49p) and calculation of mean fluorescence intensities with standard error of the mean (SEM).

### 2.5 Gene silencing

Cell cultures were transfected with siRNA sequences that are known to silence HoxA10, HoxB4, HoxB7, HoxB9, HoxD8, HoxD9, or BMP4 (Dharmacon; Supplementary material online, *Table S2*) using the Lipofectamine^®^ RNAiMAX transfection system (13778-150, Invitrogen) following the manufacturer’s instructions. Non-targeting scrambled sequences were used as a control (D-001810-01-50 ON-TARGETplus Non-targeting siRNA#1, Dharmacon).

### 2.6 Comparative real-time PCR

RNA was extracted using the RNeasy Mini Kit (74104, Qiagen) and reverse transcribed into cDNA using the iScript cDNA synthesis kit (1708891, Bio-Rad). The levels of human or porcine transcripts were assessed using qRT-PCR using gene-specific primers (Supplementary material online, *Table S3*). Reactions were prepared using SsoAdvanced universal SYBR^®^Green supermix (172-5271, Bio-Rad) and were run in triplicate following the manufacturer’s instructions. Relative gene expression was calculated by comparing the number of thermal cycles that were necessary to generate threshold amounts of product. Fold changes were calculated using the ΔΔCt method. Data were pooled from at least three independent experiments and mean values were calculated with SEM.

### 2.7 Western blotting

Total cell lysates were isolated using lysis buffer (containing 2% SDS, 10% glycerol, and 5% β-mercaptoethanol). Western blotting was carried out using specific primary antibodies (Supplementary material online, *Table S1*) and horse radish peroxidase-conjugated secondary antibodies obtained commercially from Dako and chemiluminescent detection was carried out using ECL Prime^®^ (GE Healthcare). Membranes were imaged using the Gel Doc XR+ system (Bio-Rad).

### 2.8 Assay of NF-κB DNA binding

Binding of RelA and p50 NF-κB subunits to DNA was quantified using the TransAm system according to the manufacturer’s instructions (Active Motif).

### 2.9 Immunofluorescent staining of cultured EC

Immunofluorescent staining of cultured EC was carried out using specific antibodies followed by widefield fluorescence microscopy (LeicaDMI4000B). HUVEC were fixed with paraformaldehyde (4%) and permeabilized with Triton X-100 (0.1%). Following blocking with goat or donkey serum for 30 min monolayers were incubated for 16 h with primary antibodies against proliferating cell nuclear antigen (PCNA) (proliferation marker), active caspase-3 (apoptosis marker), CDH5 (endothelial marker), RelA, HoxB9, and AlexaFluor488- or Alexafluor568-conjugated secondary antibodies. Nuclei were identified using the DNA-binding probe DAPI (Sigma). Image analysis was performed using Image J software (1.49p) to calculate the frequency of positive cells. Isotype controls or omission of the primary antibody was used to control for non-specific staining.

### 2.10 Statistical analysis

Differences between samples were analysed using an unpaired or paired Student’s *t*-test or ANOVA (*P < 0.05, **P < 0.01, ***P < 0.001).

## 3. Results

### 3.1 Low WSS induces multiple Hox genes in atheroprone endothelium

**Figure 1:**
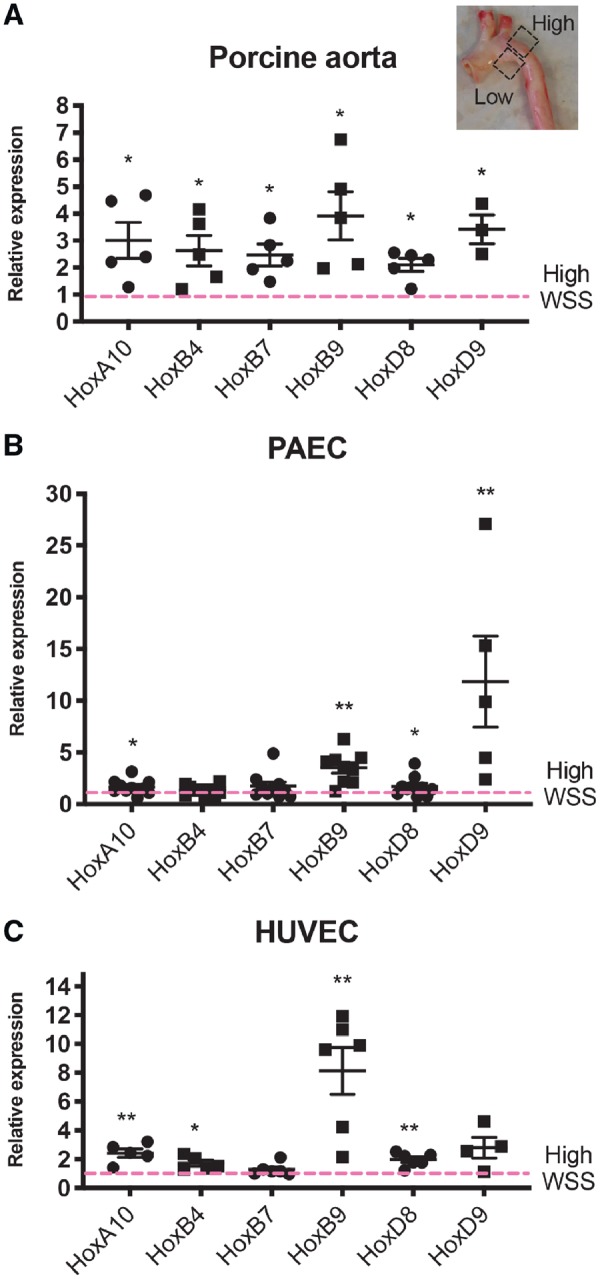
Low WSS induces multiple Hox genes. (*A*) Porcine aortae were cut along the outer curvature to expose the lumen and ECs were isolated from low and high WSS regions. Data were pooled from five pigs. (*B*) PAECs (HOXA10, HOXB4, HOXB7, HOXB9, HOXD8, *N* = 9; HOXD9, *N* = 4) or (*C*) HUVECs (HOXA10, HOXB4, *N* = 5; HOXB7, HOXB9, HOXD8, *N* = 6; HOXD9, *N* = 4) were cultured in 6-well plates and exposed to orbital shaking to generate WSS. After 72 h, cells were isolated from the centre (low WSS) or the periphery (high WSS) of the well. (*A, B*, and *C*) Hox gene mRNA levels were quantified by qRT-PCR using gene-specific primers. Mean expression levels in low WSS conditions were normalized to levels in high WSS (dotted line) and are shown with standard deviations. *P < 0.05, **P < 0.01 using an unpaired two-tailed *t*-test.

A recent microarray study from our group suggested that several Hox genes were differentially expressed at high and low WSS regions of the aorta. qPCR studies of an independent cohort of pigs demonstrated that EC isolated from the low shear stress region (inner curvature) had enriched expression of HoxA10, HoxB4, HoxB7, HoxB9, HoxD8, and HoxD9 (*Figure 1A*), thus confirming anatomical variation in Hox gene expression.

Given their enrichment at atheroprone sites, we hypothesized that Hox genes are regulated by WSS. This was tested by exposing PAEC or HUVEC to high (12 dynes/cm^2^) or low (4 dynes/cm^2^) WSS using either orbital or parallel plate flow systems. To validate the approach, we confirmed using each of these systems that CCL2 was enriched in cells exposed to low WSS whereas protective mRNAs (KLF2 or eNOS) were enhanced by high WSS (Supplementary material online, *Figure S1*). qRT-PCR revealed that mRNA levels of HoxA10, HoxB9, HoxD8, and HoxD9 were significantly elevated in PAEC exposed to low WSS compared to cells exposed to high WSS using an orbital shaker system, with HoxB9 and HoxD9 exhibiting the greatest enrichment (*Figure 1B*). A similar pattern of expression was observed in HUVEC exposed to low vs. high WSS using either an orbital (*Figure 1C*) or parallel plate system (Supplementary material online, *Figure S2*). We concluded that low WSS induces several Hox genes in atheroprone endothelium and that HoxB9 and HoxD9 were prominently expressed.

### 3.2 Hox genes drive inflammation and endothelial turnover in low shear conditions

**Figure 2:**
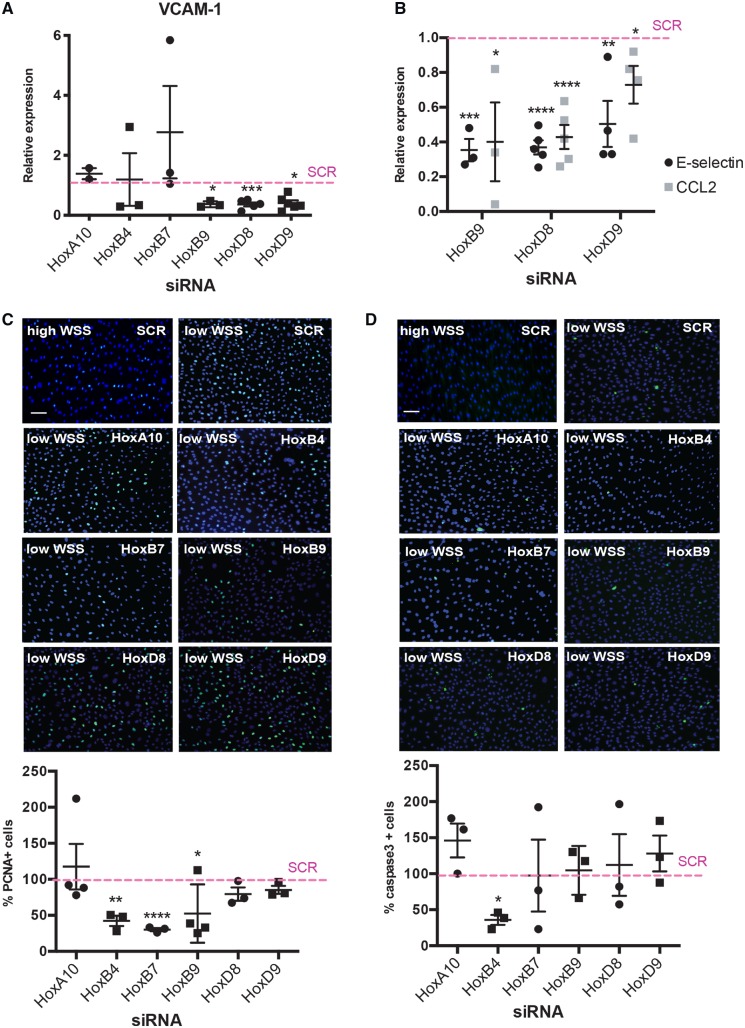
Hox genes are required for endothelial activation, proliferation, and apoptosis. HUVECs were transfected with siRNA targeting specific Hox genes or with scrambled control sequences. Cells were then exposed for 72 h to low (centre) or high (periphery) WSS using the orbital system. mRNA levels of VCAM-1 (*A*) or E-selectin and CCL2 (*B*) were quantified by qRT-PCR. Mean expression levels in low WSS conditions were normalized to levels in high WSS (dotted line) and are shown with standard deviations. Proliferation and apoptosis were quantified by immunofluorescent staining using antibodies that detect PCNA (*C*; green) or cleaved caspase-3 (*D*; green) and counterstaining nuclei using DAPI (blue). Mean frequencies of PCNA-positive or apoptotic cells in low WSS conditions were normalized to levels in high WSS (dotted line) and are shown with standard deviations. Bar = 75 μm. *P < 0.05, **P < 0.01, ***P < 0.001 using an unpaired two-tailed *t*-test.

We next studied the potential role of WSS-regulated Hox genes in inflammation, proliferation, and apoptosis. Each Hox gene was targeted in EC exposed to WSS using siRNA and the efficiency of gene silencing was measured by qRT-PCR (Supplementary material online, *Figure S3*). We used VCAM-1 expression in cultured HUVEC as a marker of inflammation. Silencing of HoxB9, HoxD8, and HoxD9 significantly reduced VCAM-1 mRNA levels under low WSS whereas HoxA10, HoxB4, and HoxB7 silencing had no effect (*Figure 2A*). To validate these observations, the regulation of VCAM-1 by HoxB9, HoxD8, and HoxD9 was confirmed using a different siRNA (Supplementary material online, *Figure S4*). Moreover, each of these Hox genes was also shown to positively regulate the expression of two other inflammatory genes, E-selectin and CCL2 (*Figure 2B*). It was concluded that HoxB9, HoxD8, and HoxD9 enhance inflammatory activation of EC exposed to pro-atherogenic disturbed flow.

Proliferation and apoptosis, which were quantified by immunofluorescent staining of PCNA or active caspase-3, were enhanced in HUVEC exposed to low compared to high WSS (Supplementary material online, *Figure S5*). Proliferation rates were significantly reduced by silencing of HoxB4, HoxB7, and HoxB9 but unaltered by silencing of HoxA10, HoxD8, and HoxD9 (*Figure 2C*). Silencing of HoxB4 reduced apoptosis (*Figure 2D*). We conclude that HoxB4, HoxB7, and HoxB9 promote proliferation in EC exposed to low WSS whereas HoxB4 is pro-apoptotic. These studies show that Hox genes have non-redundant pathophysiological roles in vascular endothelium with several promoting inflammation and others regulating proliferation and apoptosis (*Figure 6*). HoxB9 is of considerable interest because it is highly abundant at the atheroprone region of the aorta and it drives two processes that promote atherosclerosis; inflammation and proliferation.

### 3.3 HoxB9 drives inflammation at atheroprone areas

**Figure 3:**
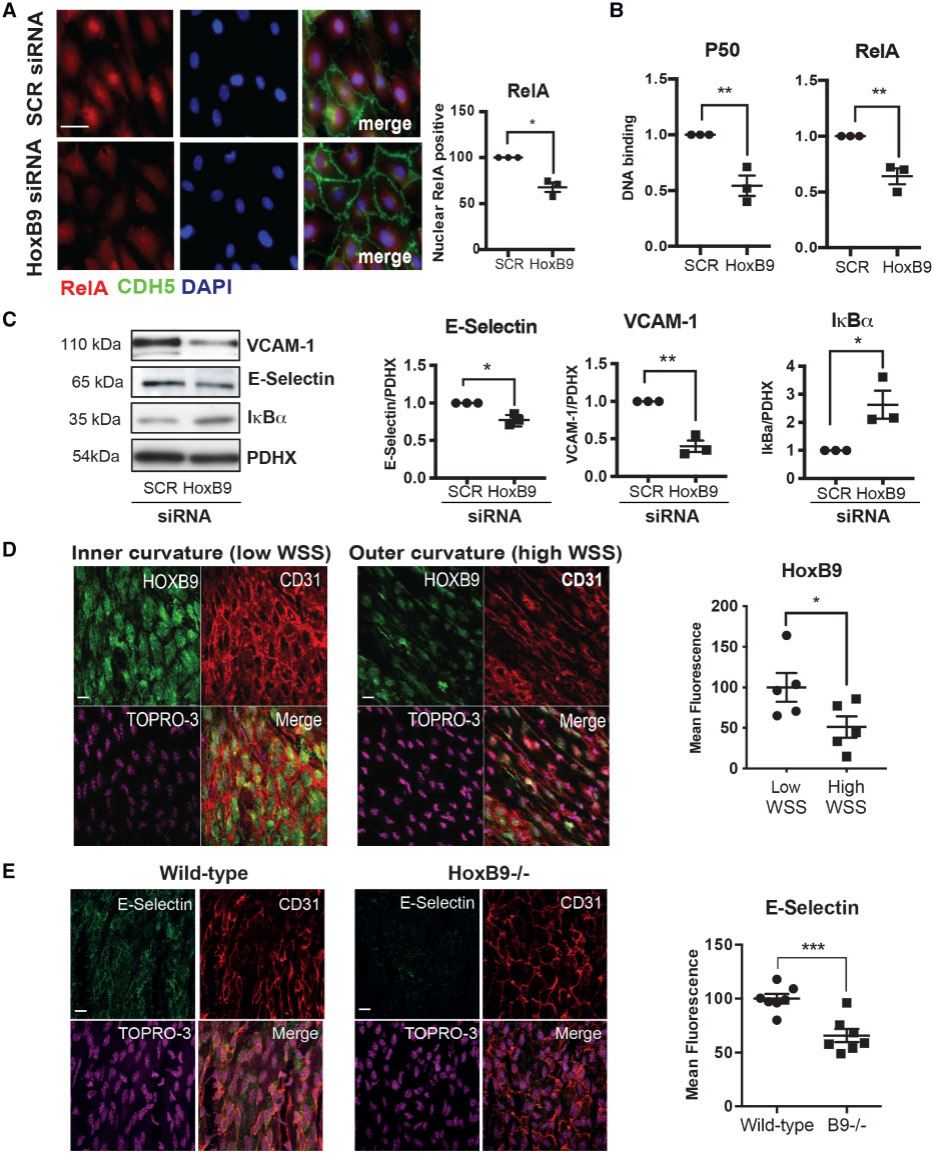
HoxB9 promotes inflammatory activation in response to low WSS. (*A–C*) HUVECs were transfected with siRNA targeting HoxB9 or with scrambled control sequences. Cells were then exposed for 72 h to low (centre) WSS using the orbital system. (*A*) Nuclear expression of RelA was assessed by immunostaining (in red) in combination with stainings for nuclei (DAPI-blue) and endothelial marker CDH5 (green). Bar = 10 μm. (*B*) The activity of RelA and p50 NF-κB subunits was assessed by DNA-binding ELISA. Mean optical density (OD) 450 nm values ± SEM are shown. (*C*) The expression levels of VCAM-1 and IκBα were assessed by Western blotting using specific antibodies and anti-Calnexin was used to control for total protein levels. (*D*) The expression of HoxB9 was quantified at the inner curvature (low WSS) and outer curvature (high WSS) of the murine aortic arch by *en face* staining (green). *N* = 5. Bar = 10 μm. (*E*) Wild-type or HoxB9^−/−^ mice were studied (*N* = 7/group). The expression of E-selectin was quantified at the inner curvature of the murine aortic arch by *en face* staining. Bar = 10 μm. (*D* and *E*) EC were identified using anti-CD31 antibodies (red) and nuclei were co-stained using TOPRO3 (purple). Differences between means were analysed using an unpaired (*A, B, C,* and *E*) or paired (*D*) *t*-test. *P < 0.05, **P < 0.01, ***P < 0.001.

To define the mechanism linking HOXB9 to inflammation we studied its potential effects on NF-κB transcription factors which induce inflammation by activating inflammatory genes. The most abundant form of NF-κB in EC is the RelA/p50 heterodimer which is enriched at atherosusceptible regions of arteries.^28^^,^^29^ Focusing on this form, we observed that silencing of HoxB9 reduced nuclear localization (*Figure 3A*) and DNA binding (*Figure 3B*) of NF-κB and simultaneously reduced the expression of VCAM-1 and E-Selectin (*Figure 3C*). It was therefore concluded that HoxB9 is a positive regulator of NF-κB. Although KLF2 can influence NF-κB,^30^ we observed that KLF2 expression was not altered by silencing of HoxB9 (Supplementary material online, *Figure S6A*). Silencing of HoxB9 enhanced the expression of the NF-κB inhibitor IκBα (*Figure 3C*), suggesting that HoxB9 activates NF-κB by reducing IκBα expression.

We validated the role of HoxB9 in inflammation by studying the expression and function of HoxB9 in EC exposed to low WSS (inner curvature) or high WSS (outer curvature) of the murine aortic arch. *En face* staining using anti-HoxB9 antibodies demonstrated that HoxB9 was significantly enriched at the low WSS region (*Figure 3D*), which is consistent with our *in vitro* observations. To study the function of HoxB9, we analysed the effects of genetic deletion of HoxB9 on endothelial activation. *En face* staining revealed that the expression of E-selectin at the low WSS region was significantly reduced in HOXB9^−/−^ compared to wild-type mice (*Figure 3E*), indicating that HOXB9 enhances inflammation at low WSS regions of arteries.

### 3.4 HoxB9 potentiates the TNF pathway

**Figure 4:**
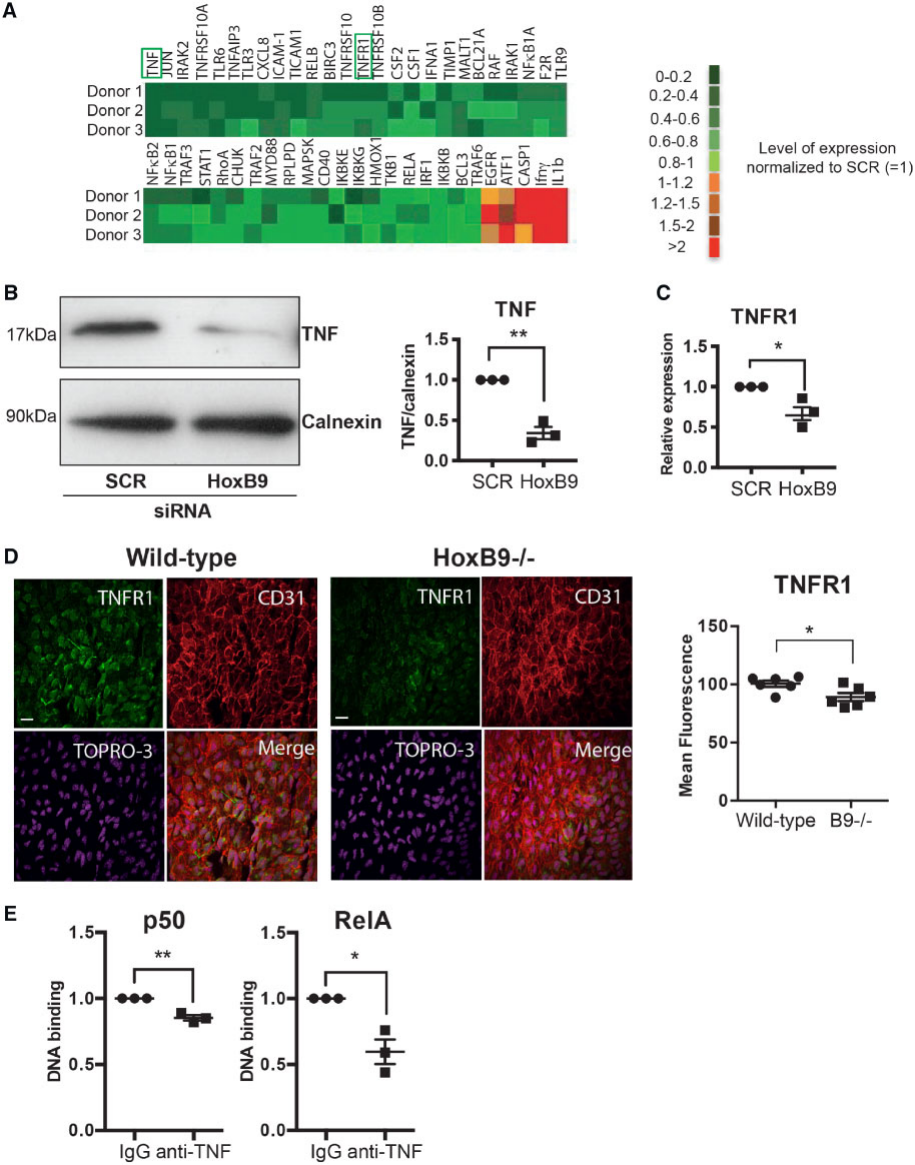
HoxB9 is required for inflammation at atheroprone regions of the aorta. (*A–C*) HUVECs were transfected with siRNA targeting HoxB9 or with scrambled control sequences. Cells were then exposed for 72 h to low WSS using the orbital system. (*A*) The expression levels of multiple transcripts that regulate inflammation were quantified by qRT-PCR array. (*B*) The expression levels of TNF were assessed by Western blotting using specific antibodies and anti-Calnexin was used to control for total protein levels. (*C*) TNFR1 mRNA levels were quantified by qRT-PCR using gene-specific primers. *N* = 3. Mean expression levels are shown with standard deviations. (*D*) Wild-type or HoxB9^−/−^ mice were studied (*N* = 6/group). The expression of TNFR1 (green) was quantified at the inner curvature of the murine aortic arch by *en face* staining. EC were identified using anti-CD31 antibodies (red) and nuclei were co-stained using TOPRO3 (purple). (*E*) HUVECs were treated with TNF blocking antibodies and then exposed for 72 h to low (centre) or high (periphery) WSS using the orbital system. The activity of RelA and p50 NF-κB subunits was assessed by DNA-binding ELISA. Mean optical density (OD) 450 nm values ± SEM are shown. *N* = 3 independent experiments. Differences between means were analysed using an unpaired *t*-test. *P < 0.05, **P < 0.01.

The mechanism linking HoxB9 to the NF-κB signalling pathway was studied in an unbiased way using a qRT-PCR array. Silencing of HoxB9 significantly reduced the expression of multiple inflammatory receptors and signalling molecules including TNF receptor superfamily members, TLRs, TRAFs, and IRAKs suggesting that it primes EC for enhanced responses to multiple inflammatory mediators (*Figure 4A* and Supplementary material online, *Figure S7*). Interestingly, HoxB9 negatively regulates the expression of IL-1β which can have both protective and pathogenic effects in atherosclerosis.^31^ We subsequently focused on the TNF pathway because HoxB9 positively regulated both TNF and its receptor TNFR1 and because this pathway is a key driver of NF-κB. Independent experiments confirmed that silencing of HoxB9 reduced the expression of TNF and TNFR1 *in vitro* (*Figure 4B and C*) and reduced TNFR1 at atheroprone areas (*Figure 4D*). Moreover, we concluded that endogenous TNF is involved in the inflammatory response to low WSS because inhibition of TNF using blocking antibodies significantly reduced NF-κB activity (*Figure 4E*). In conclusion, HoxB9 induced the expression of multiple inflammatory molecules including components of the TNF pathway which drives inflammation in response to low WSS.

### 3.5 BMP4 regulates HoxB9 expression in response to disturbed flow

**Figure 5:**
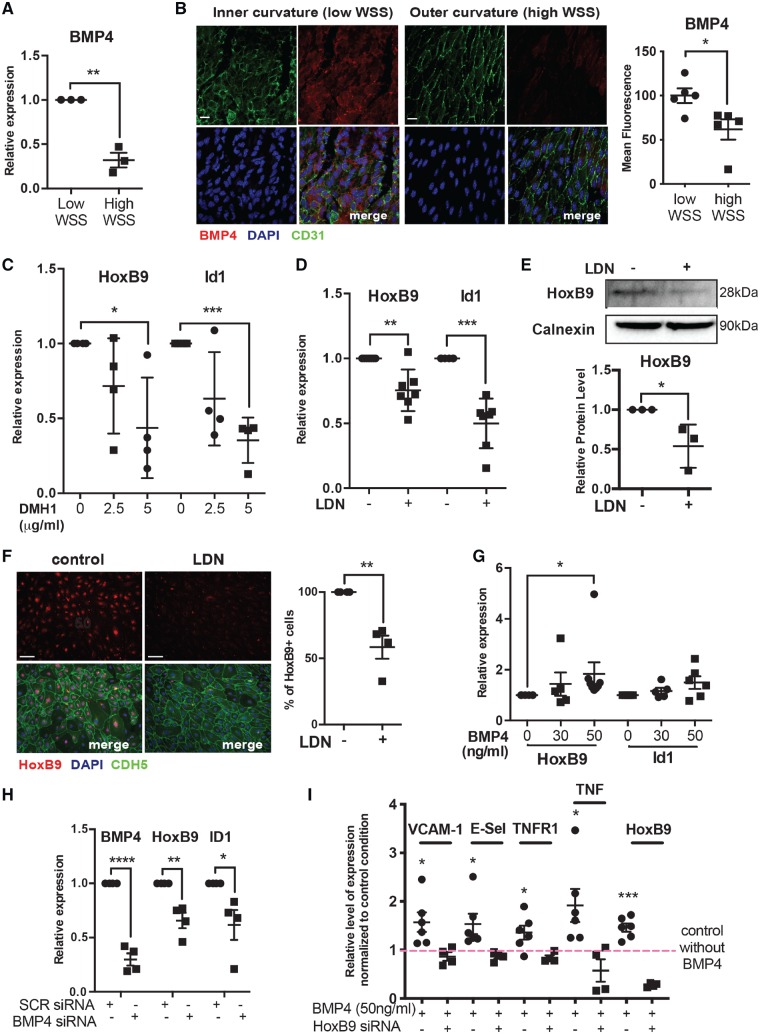
BMP4 induces HoxB9 in response to low shear stress. (*A*) HUVEC were cultured in 6-well plates and exposed to orbital shaking to generate WSS. After 72 h, cells were isolated from the centre (low WSS) or the periphery (high WSS) of the well. BMP4 mRNA levels were quantified by qRT-PCR using gene-specific primers. *N* = 3 experiments. (*B*) The expression of BMP4 was quantified at the inner curvature (low WSS) and outer curvature (high WSS) of the murine aortic arch by *en face* staining (red). EC were identified using anti-CD31 antibodies (green) and nuclei were co-stained using TOPRO3 (blue). *N* = 3. (*C–F*) HUVEC were treated with DMH1 or LDN and then exposed to low WSS for 72 h. Bar = 10 μm. (*C* and *D*) mRNA levels of HoxB9 or ID1 were quantified by qRT-PCR using gene-specific primers. *N* = 4–7 experiments. (*E*) The expression levels of HoxB9 were assessed by Western blotting using specific antibodies and anti-Calnexin was used to control for total protein levels. *N* = 4 experiments. (*F*) HoxB9 expression was assessed by immunofluorescent staining (red). EC were identified using anti-CDH5 antibodies (green) and nuclei were co-stained using DAPI (blue). *N* = 4. Bar = 50 μm. (*G*) HUVEC cultured under static conditions were treated with varying quantities of BMP4 for 72 h. mRNA levels of HoxB9 and ID1 were quantified by qRT-PCR. *N* = 6 experiments. (*H*) HUVEC were transfected with siRNA targeting BMP4 or with scrambled control sequences and then exposed for 72 h to low WSS. mRNA levels of BMP4, HoxB9, and ID1 were quantified by qRT-PCR. *N* = 4 experiments. (*I*) HUVEC cultured under static conditions were transfected with siRNA targeting HoxB9 or with scrambled control sequences, and were treated with or without BMP4. mRNA levels of inflammatory molecules and HoxB9 were quantified by qRT-PCR. *N* = 6 independent experiments. (*A–I*) *P < 0.05, **P < 0.01, ***P < 0.001 using a paired two-tailed *t*-test (*A* and *I*), unpaired two-tailed *t*-test (*D, E,* and *H*), and one-way ANOVA (*C* and *G*).

Since BMP4 is known to be enriched at low WSS regions of arteries^15^ and induces HoxB9 during embryogenesis^32^ we hypothesized that BMP4 regulates HoxB9 at atheroprone sites. Consistent with this, BMP4 is enriched in HUVEC exposed to low WSS compared to high WSS (*Figure 5A*). Similarly, *en face* staining revealed enhanced expression of BMP4 at the inner (low WSS) compared to the outer (high WSS) curvature of the murine aortic arch (*Figure 5B*). These data recapitulate previous observations that EC exposed to disturbed flow are primed for enhanced BMP signalling.^15^^,^^16^

The potential link between BMPs and HoxB9 was studied in HUVECs using DMH1 (ALK2 inhibitor) or LDN193189 (ALK1, ALK2, ALK3, ALK6 inhibitor). Both compounds reduced the expression of the BMP target gene ID1 thereby confirming their efficacy (*Figure 5C *and* D*). Inhibition of BMP signalling significantly reduced the expression of HoxB9 mRNA (*Figure 5C and D*) *and* HoxB9 protein (*Figure 5E *and* F*), demonstrating that BMP signalling positively regulates HoxB9 in response to low WSS. We next focused on the potential role of BMP4 in HoxB9 up-regulation. The addition of exogenous BMP4 to cultured HUVEC induced HoxB9 in a dose-dependent manner (*Figure 5G*), whereas silencing of BMP4 reduced HoxB9 expression in HUVEC exposed to low WSS (*Figure 5H*). We concluded that low WSS induces HoxB9 via BMP4 in vascular endothelium. Interestingly, silencing of HoxB9 significantly enhanced the expression of BMP4 indicating that HoxB9 is a negative regulator of BMP4 (Supplementary material online, *Figure S8*). Overall, these data demonstrate that BMP4 induces HoxB9 in vascular endothelium, and that a negative feedback loop exists to limit BMP4 expression.

We next investigated whether the BMP4-HoxB9 pathway promotes inflammation by quantifying inflammatory gene expression in HUVEC that were treated with BMP4 in the presence of absence of HoxB9. It was observed that BMP4 induced TNF and TNFR1, and also induced VCAM-1 and E-selectin, and that these responses were abolished by silencing of HoxB9 (*Figure 5I*). By contrast, BMP4 did not alter the expression of KLF2 (Supplementary material online, *Figure S6B*). Therefore, it was concluded that BMP4 promotes inflammatory gene expression via HoxB9.

Finally, we investigated whether the BMP4-HoxB9-TNF axis is potentially involved in atherogenesis by determining the effects of hypercholesterolaemia on the expression of components of this pathway. Hypercholesterolaemia was induced in ApoE^−/−^ mice by exposure to a high fat diet for 6 weeks. *En face* staining of the aorta revealed that the expression of BMP4, HoxB9, and TNFR1 at the inner curvature (low WSS) was enhanced in hypercholesterolaemic mice compared to controls (Supplementary material online, *Figure S9*), indicating that the BMP4-HoxB9-TNF pathway is potentiated by pro-atherogenic conditions.

In conclusion, low WSS and hypercholesterolaemia induce a BMP4-HoxB9-TNF-NF-κB pathway that drives inflammation during early atherogenesis.

## 4. Discussion

### 4.1 A Hox gene code regulates EC fate at disease-prone sites

Hox genes are critical developmental genes involved in the patterning of the embryos along their anterior–posterior and proximal–distal axis. However, Hox genes are also expressed in adult, notably in the vasculature where several of them are differentially expressed at atheroprone and atheroprotected regions of the arterial tree.^20–26^^,^^33^ For example, HoxA9, HoxA10, and HoxD3 were expressed at higher levels in EC isolated from iliac arteries that are relatively protected from atherosclerosis compared to coronary arteries that are atherosusceptible.^20^^,^^21^ Moreover, HoxA1 endothelial expression is enhanced in the aortic arch (atherosusceptible) compared to the thoracic aorta (atheroprotected)^22^ whereas HoxA4 displayed the opposite pattern.^23^ Collectively, these observations suggest that the spatial organization of HOX gene expression in EC may influence focal atherosusceptibility.

An elegant study from Trigueros-Motos *et al.*^26^ revealed that the expression of Hox genes in vascular smooth muscle cells (VSMCs) also varies according to anatomy. Intriguingly, the expression of HoxA10, HoxB7, HoxB9, HoxD8, and HoxD9 in VSMCs was reduced in the aortic arch compared to the thoracic aorta,^26^ whereas the expression of these Hox genes in EC was enhanced at the inner curvature of the aortic arch. Trigueros-Motos *et al.*^26^ suggested that regional differences in Hox gene expression in VSMCs may reflects the divergent embryological origins of VSMC at these sites which arise from neuroectoderm (aortic arch) and paraxial mesoderm (thoracic aorta). By contrast, EC originate from paraxial mesoderm throughout the aorta and it is therefore likely that regional differences in Hox gene patterns are generated by environmental factors such as local shear stress conditions. The observation that Hox genes are co-expressed in atheroprone EC and atheroprotected VSMCs has implications for therapeutic targeting of these molecules which should be directed specifically to the endothelial pool, e.g. using siRNA-nanoparticles.^34^

**Figure 6:**
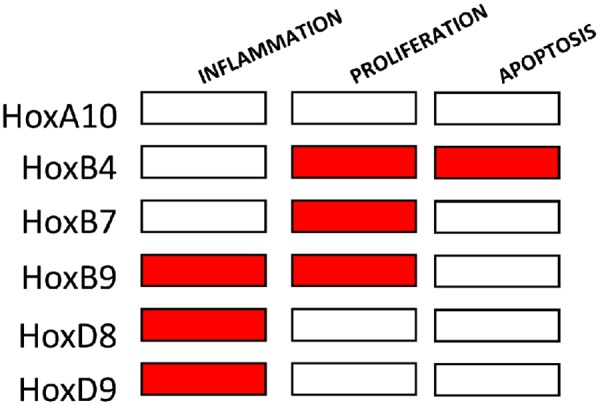
Summary of Hox gene functions in atheroprone endothelium. HoxA10, HoxB4, HoxB7, HoxB9, HoxD8, and HoxD9 were tested for regulation of inflammation, proliferation or apoptosis in EC exposed to low WSS. Red box indicates positive regulation.

Hox genes influence angiogenesis by controlling the expression of integrins, MMPs, ephrins, and other molecules,^35^ however, their functional significance in adult EC is largely unknown. Here we used gene silencing to study the potential role of Hox genes in regulating inflammation, proliferation, and apoptosis in EC exposed to low WSS. Our study revealed that HoxB4, HoxB7, and HoxB9 were positive regulators of EC proliferation, and that HoxB9, HoxD8, and HoxD9 had a pro-inflammatory function. EC apoptosis was positively regulated by HoxB4. We conclude that Hox genes regulate several pathophysiological functions in disease prone regions of arteries. Most of the Hox genes that we studied had distinct functional profiles with differing effects on inflammation, proliferation, and apoptosis (*Figure 6*). Therefore, we propose that the expression pattern of multiple Hox genes acts as a code that controls the fate of EC by inducing activation, proliferation, or apoptosis. Further work is required to understand the mechanisms that EC use to decode Hox gene expression profiles and convert this information into physiological responses.

### 4.2 A BMP4-HoxB9-TNF pathway primes inflammation at disease-prone sites

We focused our studies on HoxB9 because inhibiting this Hox gene could be particularly effective in treating atherosclerosis since it drives two distinct pathophysiological functions, inflammation and proliferation. Previous studies revealed that HOXB9 is essential for specification of the thoracic skeleton^27^ and forelimbs.^36^ Here we demonstrate that in addition to its roles in the patterning of organs during development, HoxB9 also plays a role in the patterning of arterial inflammation. We investigated the mechanism and observed by qRT-PCR array that HoxB9 regulated the expression of multiple inflammatory molecules including cytokines and other mediators (e.g. TNF, CD40), receptors (e.g. TNFR1, TLRs), and signalling molecules and transcription factors (e.g. NF-κB, TRAFs, IRAKs). Interestingly, Passerini *et al.*^6^ found that low WSS primes EC for enhanced inflammation by increasing the expression of multiple cytokines, receptors, and signalling molecules belonging to several different pathways. Here we provide a mechanism for inflammatory priming at the low WSS site by demonstrating that HoxB9 is a high-level regulator of the expression of multiple inflammatory components. We focused our validation studies on the TNF-TNFR1 pathway and confirmed that it is up-regulated by HoxB9 and required for NF-κB activation by low WSS. Further work should now be carried out to determine the role of HoxB9 in regulating other inflammatory pathways including the IL-1/TLR^35^ pathway in atherosclerosis.

We next investigated the potential role of BMP4 in regulating HoxB9 expression because these molecules are coupled in developmental systems.^32^ Using cultured EC and murine models we demonstrated that BMP4 is induced by low WSS and that it is a positive regulator of HoxB9 and downstream inflammatory activation. Moreover, the expression of BMP4, HoxB9, and TNFR1 at disease prone sites was increased under hypercholesterolaemic conditions. These data are consistent with previous observations that BMP4 is a driver of inflammation and atherosclerosis at low WSS sites.^14–16^ Interestingly, Sorescu *et al.*^16^ previously found that BMP4 enhances NOX1-dependent production of reactive oxygen species (ROS) which are known to enhance NF-κB activation in response to TNF. We propose therefore that BMP4 promotes vascular inflammation by activating two parallel pathways that act synergistically to activate NF-κB; HoxB9-TNF-TNFR1 signalling; and NOX1-dependent production of ROS. This concept is consistent with a well-recognized role for TNF and ROS in arterial inflammation^37^ and it positions the BMP4-HOXB9 pathway as a key upstream regulator. In addition to its role in atherosclerosis, BMP signalling is involved in the pathogenesis of other vascular diseases including pulmonary arterial hypertension^38^ and hereditary haemorrhagic telangiectasia.^39^ Therefore, further studies should now be carried out to determine the potential role of the BMP-HoxB9-TNF axis in other vascular diseases.

**Translational perspective**

Here we report that BMP4-HoxB9-TNF signalling is activated at disease-prone regions of arteries and that it promotes endothelial cell turnover and inflammation which are important initiators of atherosclerosis. The corollary is that components of this pathway that could be targeted therapeutically to prevent or treat atherosclerosis. Future studies should therefore determine whether inhibition of endothelial BMP4 or HoxB9, for example, using siRNA nanoparticles, can reduce atherosclerosis. Moreover, BMP inhibitors are currently under development for the treatment of cancer, anaemia, and bone overgrowth and could be repurposed to treat atherosclerosis.

## Supplementary material

Supplementary material is available at *Cardiovascular Research* online.

## Supplementary Material

cvz235_Supplementary_Data
