# Supplementary material for: Homeobox B9 integrates bone morphogenic protein 4 with inflammation at atheroprone sites
Source: Cardiovasc Res. 2019 Aug 29;116(7):1300–10. doi: 10.1093/cvr/cvz235 (PMC7243277; doi:10.1093/cvr/cvz235)
Supplement: cvz235_Supplementary_Data [file cvz235_supplementary_data.pdf]

Homeobox B9 integrates bone morphogenic protein 4 with inflammation at atheroprone sites.

Souilhol C, Gauci I, Feng S, Tardajos Ayllon B, Mahmoud M, Canham L, Fragiadaki M, Serbanovic-Canic J, Ridger V, Evans PC*.

Short title: HoxB9 promotes arterial inflammation

Category: Atherosclerosis and Lipid Biology

Total word count: 5876

* Address correspondence to:

Professor Paul Evans PhD, FESC, FRSB
Department of Infection, Immunity and Cardiovascular Disease,
Bateson Centre for Lifecourse Biology,
INSIGNEO Institute for Cardiovascular Medicine,
University of Sheffield,
Sheffield S10 2RX, UK.

Telephone: 0114 2159525
Email: paul.evans@sheffield.ac.uk

SUPPLEMENTARY FIGURES

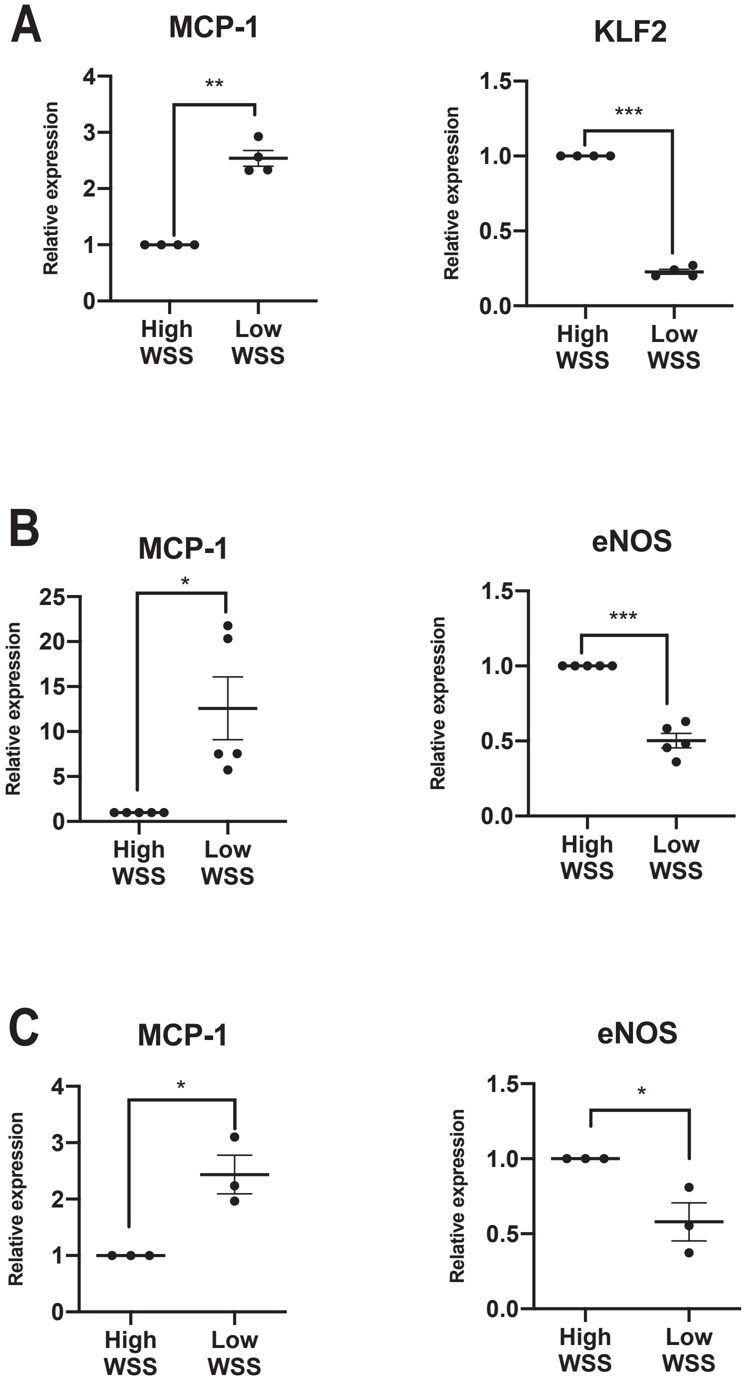

Supplementary Figure 1. Validation of endothelial responses to shear stress. (A) PAECs or (B) HUVECs were cultured in 6-well plates and exposed to orbital shaking to generate WSS. After 72 h, cells were isolated from the centre (low WSS) or the periphery (high WSS) of the well. (C) HUVEC were exposed to low or high WSS for 72 h using a parallel-plate system. (A-C) mRNA levels of CCL2, KLF2 or eNOS were quantified by qRT-PCR. Data were pooled from 4 (A), 5 (B) or 3 (C) experiments and mean expression levels are shown with standard deviations. *p<0.05, **p<0.01, ***p<0.001 using a paired two-tailed t-test.

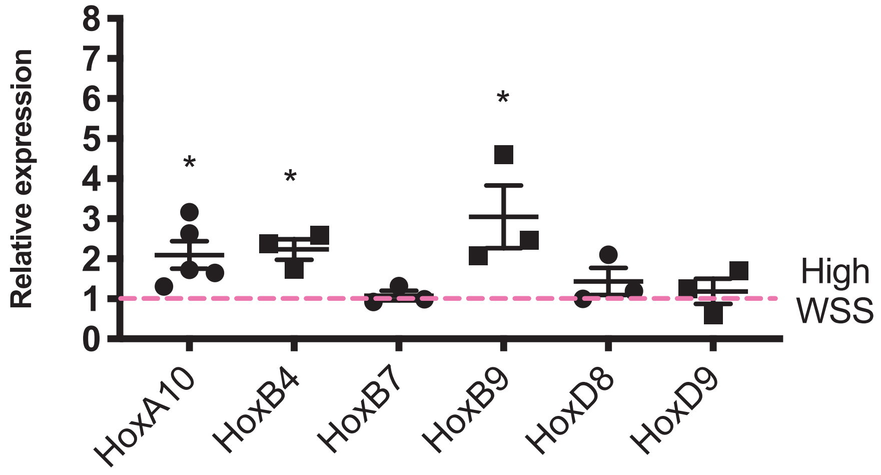

Supplementary Figure 2. Low WSS generated using a parallel-plate system induces multiple Hox genes. HUVEC were exposed to low or high WSS for 72 h using a parallel-plate system. After 72 h, Hox gene mRNA levels were quantified by qRT-PCR using gene-specific primers. Data were pooled from 5 independent experiments. Mean expression levels in low WSS conditions were normalised to levels in high WSS (dotted line) and are shown with standard deviations. *p<0.05, **p<0.01 using a paired two-tailed t-test.

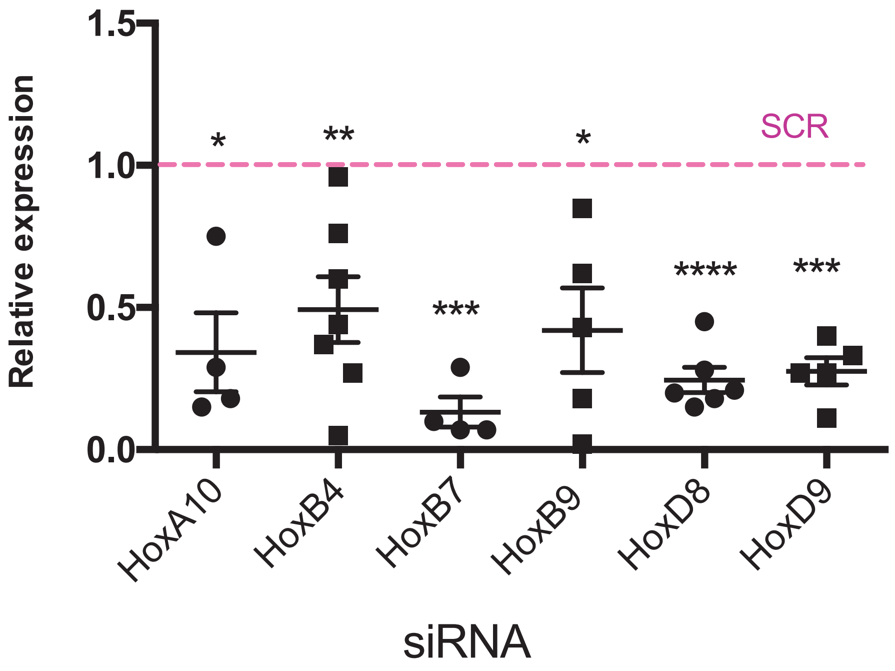

Supplementary Figure 3. Validation of Hox gene silencing.
HUVECs were transfected with siRNA targeting specific Hox genes or with scrambled control sequences. Cells were then exposed for 72 h to low WSS using the orbital system. Hox gene mRNA levels were quantified by qRT-PCR using gene-specific primers (HoxA10, N=4; HoxB4, N=7; HoxB7, N=4; HoxB9, N=5; HoxD8, N=6; HoxD9, N=5). Mean expression levels in cells treated with Hox-specific siRNAs were normalised to levels in scrambled controls (dotted line) and are shown with standard deviations. *p<0.05 using a paired two-tailed t-test.

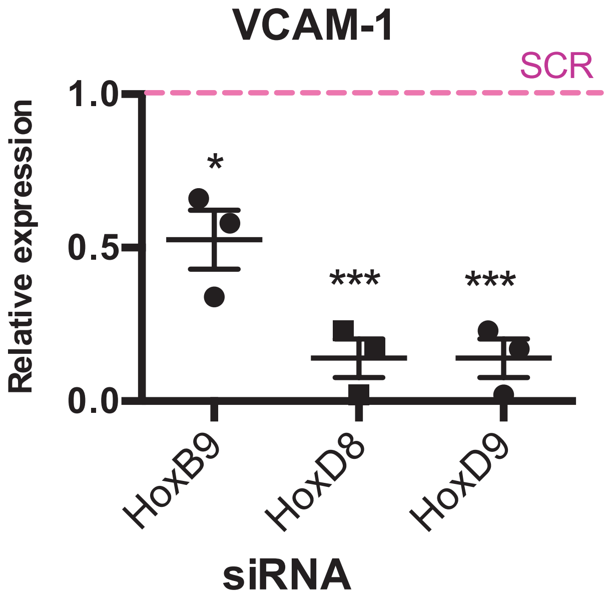

Supplementary Figure 4. Silencing of HoxB9, HoxD8 or HoxD9 using a second siRNA sequence reduced VCAM-1 expression.

HUVECs were transfected with siRNA targeting HoxB9, HoxD8 or HoxD9 or with scrambled control sequences. Cells were then exposed for 72 h to low WSS using the orbital system. VCAM-1 mRNA levels were quantified by qRT-PCR. Data were pooled from 3 experiments. Mean expression levels in cells treated with Hox-specific siRNAs were normalised to levels in scrambled controls (dotted line) and are shown with standard deviations. *p<0.05, ***p<0.001 using a paired two-tailed t-test.

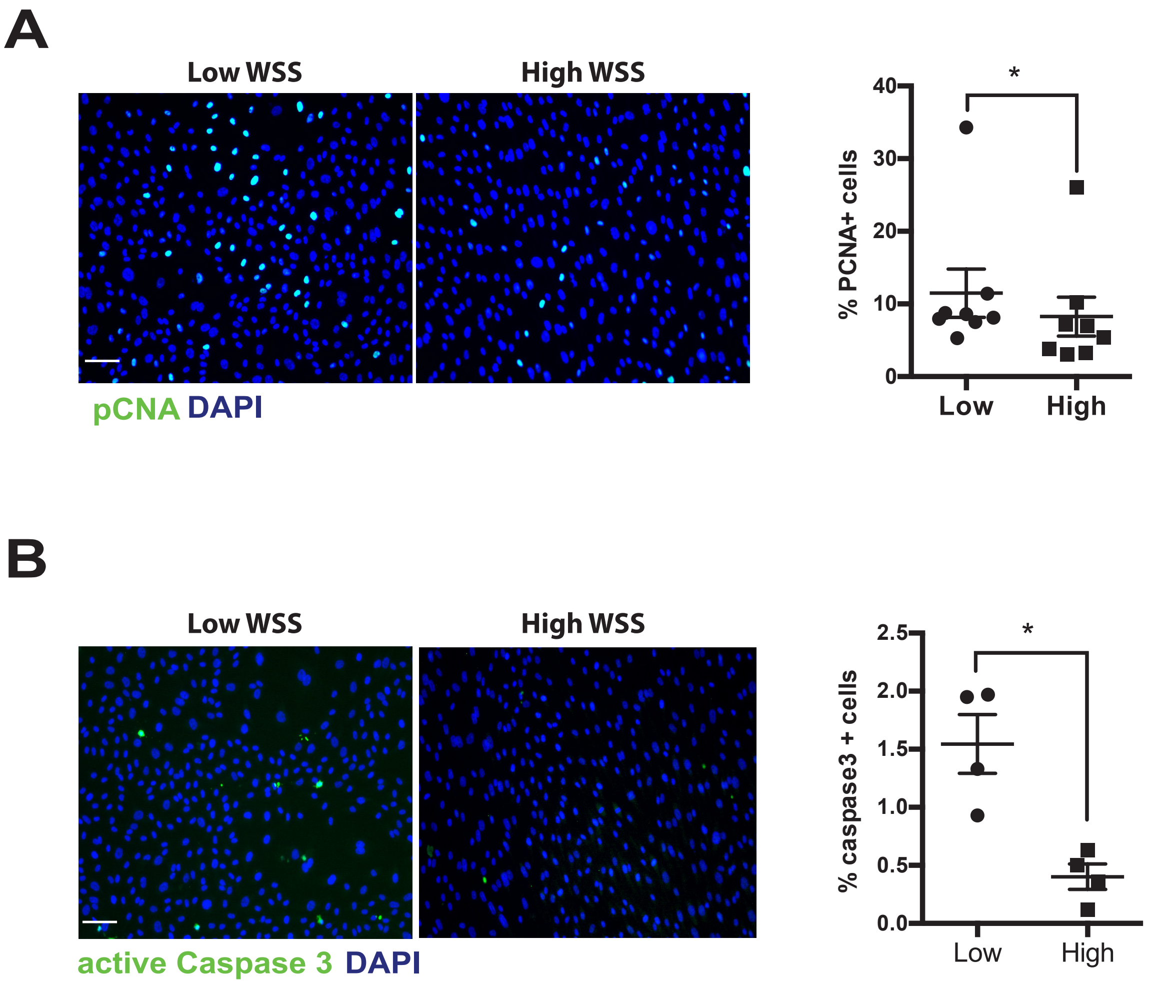

Supplementary Figure 5. Low shear stress enhanced proliferation and apoptosis in cultured endothelial cells.
HUVEC were cultured in 6-well plates and exposed to orbital shaking to generate low or high WSS. Proliferation and apoptosis were quantified by immunofluorescent staining using antibodies that detect PCNA or cleaved caspase-3. Bar = 75μm. Frequencies of PCNA-positive (A; N=8 experiments) or apoptotic cells (B; N=4 experiments) were pooled and mean values are shown with standard deviations. *p<0.05 using a paired two-tailed t-test.

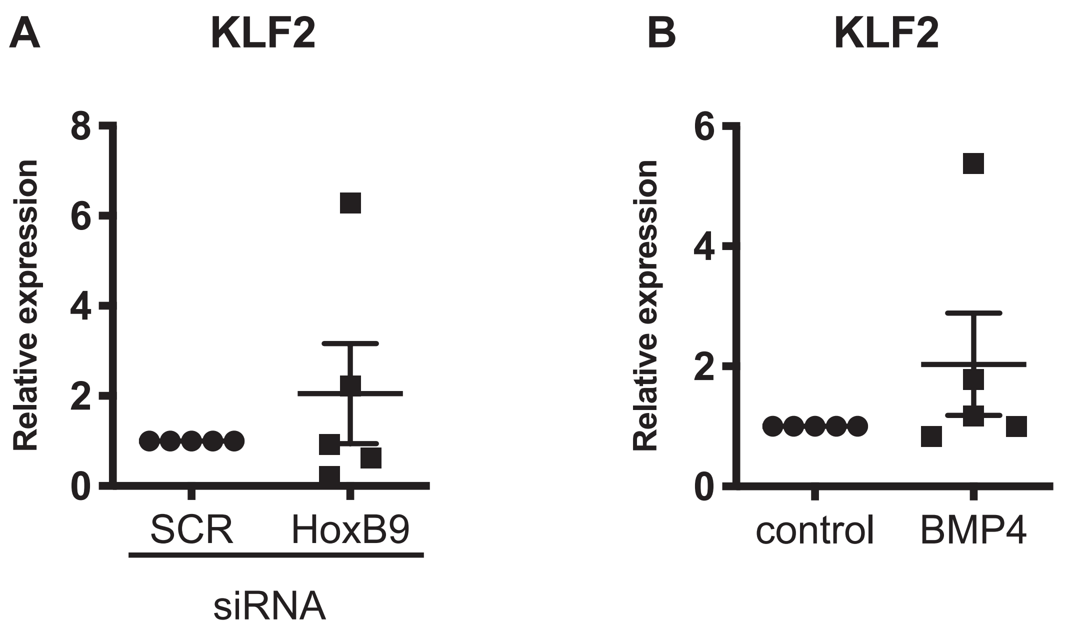

Supplementary Figure 6: Effect of HoxB9 and BMP4 on KLF2 expression. (A) HUVEC were transfected with siRNA targeting HoxB9 or with scrambled control sequences and then exposed for 72 h to low WSS. (B) HUVEC cultured under static conditions were treated with 50ng/ml of BMP4 for 72 h. (A, B) mRNA levels of KLF2 was quantified by qRT-PCR. Data were pooled from 5 experiments and mean expression levels are shown with standard deviations.

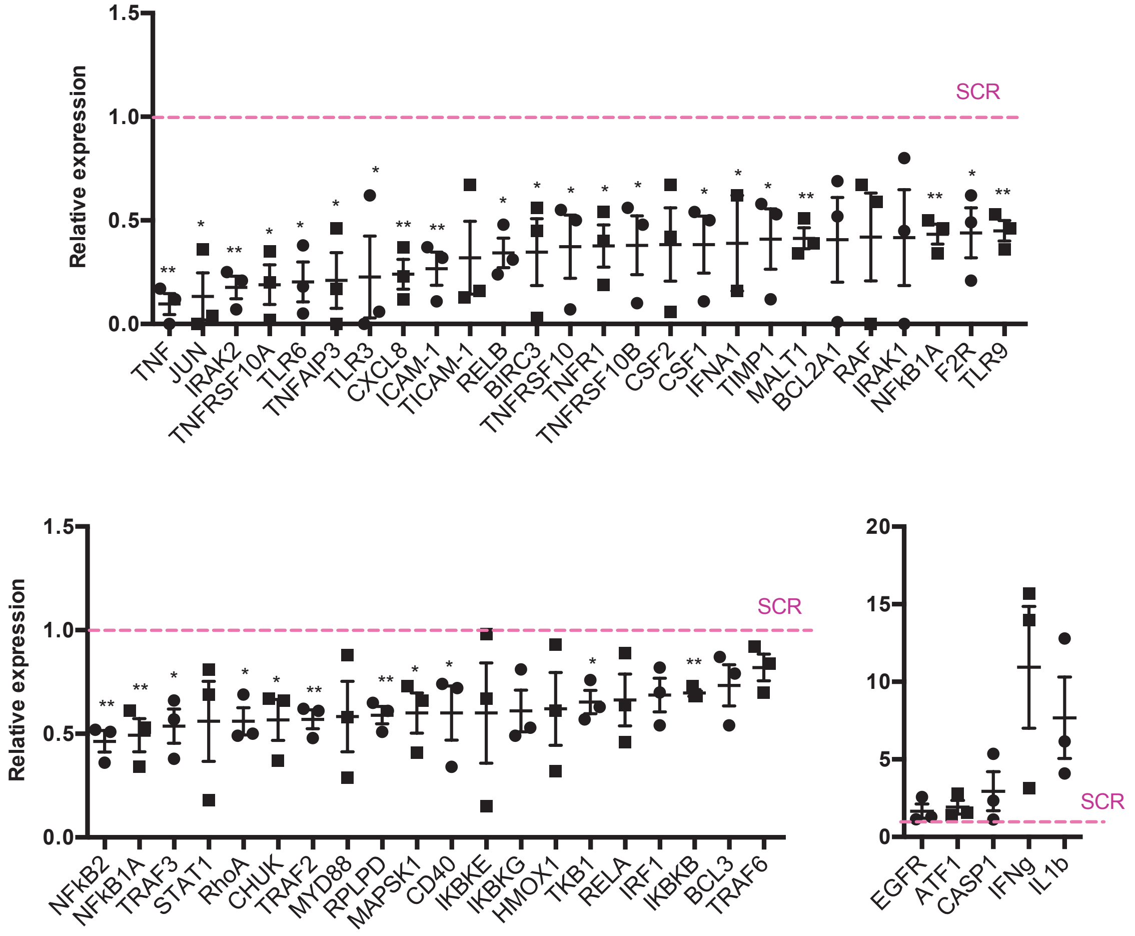

Supplementary Figure 7: HoxB9 positively regulates multiple pro-inflammatory molecules. HUVECs were transfected with siRNA targeting HoxB9 or with scrambled control sequences. Cells were then exposed for 72 h to low WSS using the orbital system. The expression levels of multiple transcripts that regulate inflammation were quantified by qRT-PCR array. Fold changes for inflammatory molecules between scramble or HoxB9 siRNA treated HUVEC are shown. *$p<0.05$, **$p<0.01$ using a paired two-tailed t-test.

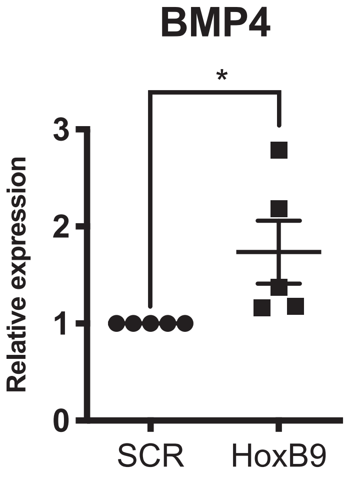

Supplementary Figure 8: Effect of HoxB9 knock-down on BMP4 expression. HUVEC were transfected with siRNA targeting HoxB9 or with scrambled control sequences and then exposed for 72 h to low WSS. mRNA levels of BMP4 was quantified by qRT-PCR. Data were pooled from 5 experiments and mean expression levels are shown with standard deviations.

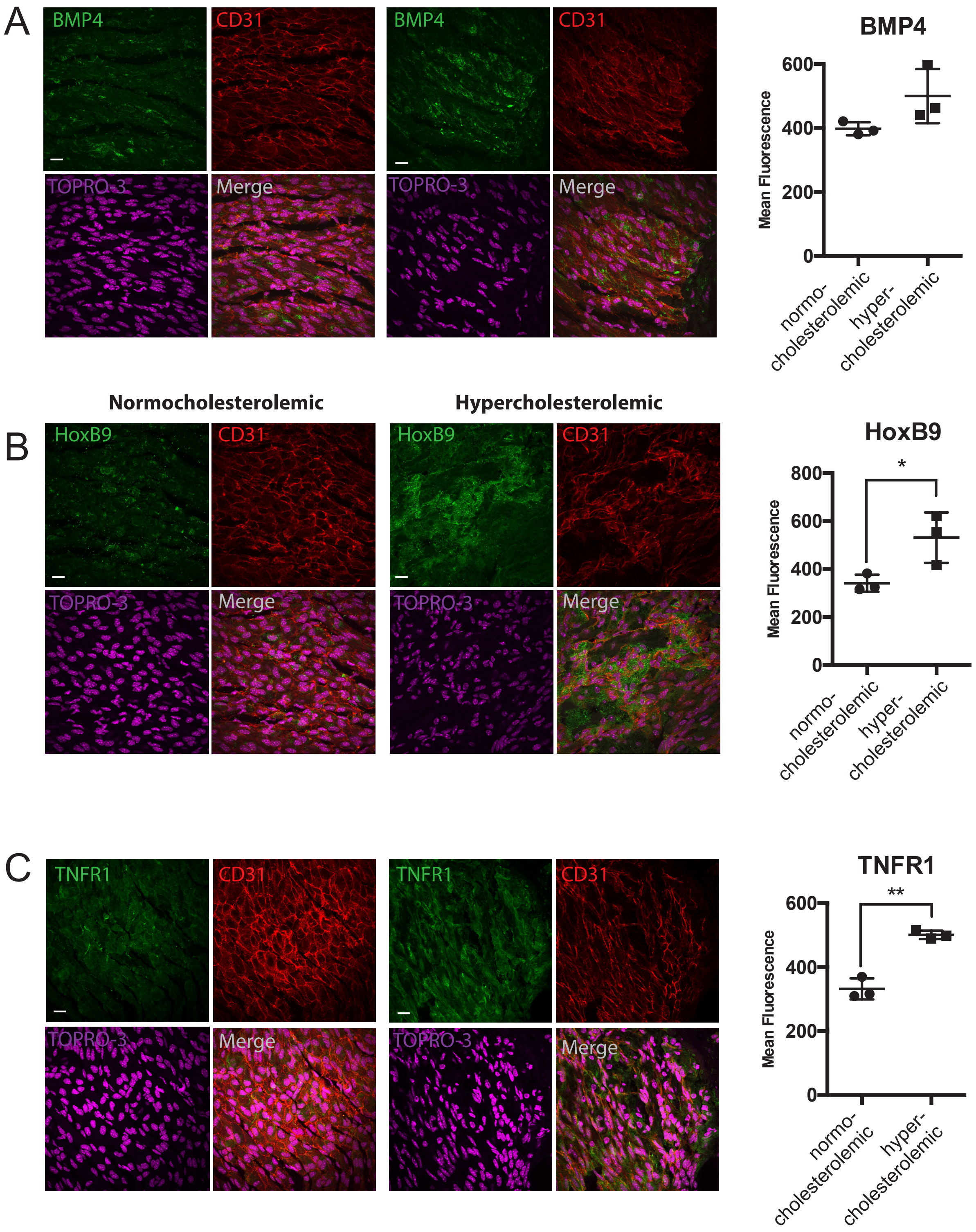

Supplementary Figure 9: Hypercholesterolemia potentiates the BMP4-HoxB9-TNF pathway at atheroprone sites. The expression of BMP4, HoxB9 and TNFR1 was quantified at the inner curvature (low WSS) of the aortic arch in normocholesterolemic (APOE $^{-/-}$ mice under normal diet) and hypercholesterolemic mice (APOE $^{-/-}$ mice under high fat diet for 6 weeks) by *en face* staining (green). EC were identified using anti-CD31 antibodies (red) and nuclei were co-stained using TOPRO3 (purple). N=3. Bar = 10μm. **p<0.01, ***p<0.001 using an unpaired two-tailed t-test.

SUPPLEMENTARY TABLES

Supplementary Table 1 Antibodies: suppliers and concentrations used.

Antibody	Company	Use	Final Concentration or dilution ratio
HOXB9	Santa Cruz sc-46129	IF	1:100
E-Selectin	Novus NBP1-45545	WB<br>IF	0.5-4μg/ml<br>2μg/ml
IκBα	Cell signaling 9242	WB	0.1μg/ml
Calnexin	BD 610524	WB	0.3μg/ml
VCAM-1	Novus NBP1-95622	WB	1:1000-10000
PDHX	Santa-Cruz sc-393644	WB	0.2μg/ml
TNFR1	Abcam ab19139	WB<br>IF	1μg/ml<br>5μg/ml
TNF	Abcam ab19139	WB<br>IF	1μg/ml<br>2μg/ml
RelA	Santa Cruz sc-372	IF	1μg/ml
CDH5	BD Bioscience 555661	IF	1μg/ml
pCNA	Abcam Ab15497	IF	5μg/ml
aCasp3	Cell Signaling 9661S	IF	2μg/ml
BMP4	Santa Cruz Sc6896	IF	1μg/ml
CD31	Biolegend 102513	IF	5μg/ml

WB, Western blotting; IF, immunofluorescence

Supplementary Table 2 siRNA sequences

Gene	Catalogue number
ON-TARGETplus human HoxA1 (3198)siRNA- smart pool	L-077464-00-0005
ON-TARGETplus human HoxA9 (3205)siRNA- smart pool	L-006337-00-0005
ON-TARGETplus human HoxA10 (3206)siRNA- smart pool	L-006336-00-0005
ON-TARGETplus human HoxB4 (3214)siRNA- smart pool	L-012892-01-0020
ON-TARGETplus human HoxB7 (3217)siRNA- smart pool	L-010515-02-0005
ON-TARGETplus human HoxB9 (3219)siRNA- smart pool	L-017548-01-0020
ON-TARGETplus human HoxD8 (3234)siRNA- smart pool	L-013244-00-0005
ON-TARGETplus human HoxD9 (3235)siRNA- smart pool	L-012494-01-0005
ON-TARGETplus human HoxB9 (3219)siRNA- individual	J-017548-09-0010
ON-TARGETplus human HoxB9 (3219)siRNA- individual	J-017548-10-0010
ON-TARGETplus human HoxB9 (3219)siRNA- individual	J-017548-11-0010
ON-TARGETplus human HoxB9 (3219)siRNA- individual	J-017548-12-0010
ON-TARGETplus human HoxD8 (3242)siRNA- individual	J-013244-05-0010
ON-TARGETplus human HoxD8 (3242)siRNA- individual	J-013244-06-0010
ON-TARGETplus human HoxD9 (3235)siRNA- individual	J-012494-11-0010
ON-TARGETplus human HoxD9 (3235)siRNA- individual	J-012494-12-0010
ON-TARGETplus human BMP4 (3219)siRNA- smart pool	L-011221-00-0005

All siRNA were purchased from Dharmacon-Horizon Discovery.

Supplementary Table 3 PCR primers.

Porcine primers:

Gene	Forward	Reverse
**HoxB4**	5’-TATGTCGACCCAAGTTCCC-3’	5’-AAGCTGCTCTCTCGCCTCT-3’
**HoxB7**	5’-TCGAGCCGAGTTCCTTCAAC-3’	5’-TC CAGGGGTAGATCCGGAA-3’
**HoxB9**	5’-TGTCCATTTCTGGGACGCTT-3’	5’-CGGAAGGAAACTTGGCTGGA-3’
**HoxD8**	5’-GCCGATTTTTACGACCCAGC-3’	5’-AGCTGCTTGTGGTCTCATCC-3’
**HoxD9**	5’-GGACTGGCTCTGGGTGTTTT-3’	5’-AAGCTTTTCCTCCCTGCCAA-3’
**KLF2**	5’-CGGCAAGACCTACACAAAGA-3’	5’-GT TGCAGTGGTAGGGCTTCT-3’

Human primers:

Gene	Forward	Reverse
**BMP4**	5’-TCCACAGCATGGTCTTGAG-3’	5’-TGGGATGTTCTCCAGATGTTCT-3’
**CCL2**	5’-GCAGAAGTGGGTTCAGGATT-3’	5’-TGGGTTGTGGAGTGAGTGTT-3’
**KLF2**	5’-AGACCACGATCCTCCTTGAC-3’	5’-ATCACAAGCCTCGATCCTCT-3’
**eNOS**	5’-CACATGGCCTTGGACTGAA-3’	5’-CAGAGCCCTGGCCTTTTC-3’
**E-Selectin**	5’-GCTCTGCAGCTCGGACAT-3’	5’-GAAAGTCCAGCTACCAAGGGAAT-3’
**HoxA10**	5’-CAAGGCAATTCCAAAGGC-3’	5’-GCTCTCGAGTAAGGTACATGTTG-3’
**HoxB4**	5’-ACGAGTCAGGGGTCGGAAT-3’	5’-CATGGAGGGAACTTGGGGTC-3’
**HoxB7**	5’-CTGGATGCGAAGCTCAGG-3’	5’-TCTTTCTCCAGCTCCAGGGTCT-3’
**HoxB9**	5’-GAGAGGCCGGATCAAACCAA-3’	5’-CTACGGTCCCTGGTGAGGTA-3’
**HoxD8**	5’-GCCGATTTTTACGACCCAGC-3’	5’-GGAGCTGCTTGTGGTCTCAT-3’
**HoxD9**	5’-GCAGCAACTTGACCCAAACAA-3’	5’-GT CCAGCTCAAGCGTCTGGTAT-3’
**ID1**	5’-CTGCTCTACGACATGAACGGC-3’	5’- AGACGTGCTGGAGAATCTCCA-3’
**VCAM-1**	5’-CATTGACTTGCAGCACCACA-3’	5’-AGATGTGGTCCCCTCATTCG-3’
